# The Coexistence of the Suprascapular Notch and Suprascapular Foramen: A Case Report

**DOI:** 10.7759/cureus.81669

**Published:** 2025-04-03

**Authors:** George Paraskevas, Christos Lyrtzis, Chrysanthos Chrysanthou, Nektarios Galanis, Nikolaos Anastasopoulos

**Affiliations:** 1 Department of Anatomy and Surgical Anatomy, Faculty of Health Sciences, Medical School, Aristotle University of Thessaloniki, Thessaloniki, GRC; 2 Department of Anatomy and Surgical Anatomy, Aristotle University of Thessaloniki, Thessaloniki, GRC; 3 Department of Orthopedics and Traumatology, Interbalkan Medical Center, Thessaloniki, GRC; 4 Department of Anatomy and Surgical Anatomy, National and Kapodistrian University of Athens School of Medicine, Athens, GRC; 5 Department of Anatomy, Aristotle University of Thessaloniki, Thessaloniki, GRC

**Keywords:** ossification, scapula, scapular notch, suprascapular foramen, suprascapular nerve neuropathy

## Abstract

Commonly, the suprascapular notch (SN), which is bridged by the superior transverse scapular ligament (STSL), carries the suprascapular nerve (SSN). Variations in the form of that notch, as well as partial or complete ossification of the STSL or other proper ligaments in the SN area, could potentially lead to SSN entrapment syndrome. The current study displays a very rare variant of coexistence of a shallow SN and a suprascapular foramen (SF) in a male scapula of unknown age. Similar cases of coexistence of an SN and an SF detected in the literature, as well as their clinical applications, are discussed.

## Introduction

As it is commonly known, the suprascapular notch (SN) of the superior border of the scapula is crossed by the superior transverse scapular ligament (STSL). Thus, the formed fibro-osseous foramen conducts the suprascapular nerve (SSN) to the supraspinatus fossa, with the suprascapular vessels passing above the STSL. The ligament mentioned above is sometimes ossified, leading to the formation of the suprascapular foramen (SF) [1]. Such ossification has been frequently seen with increasing age based on the formation of enthesophytes [2]. However, genetic factors have been suggested as responsible for STSL ossification [3]. The incidence of SF ranges in various populations, between 2% in Indians of Manipur [4] and 30.76% in Brazilians [5]. Such an SF may potentially predispose to the SSN entrapment syndrome due to the SSN impingement on the SF margin when the arm is moving.

In the current study, we present, to the best of our knowledge, a very rare variant of the co-existence of an SF with a shallow SN, discussing its potential clinical applications and the relevant literature. The association of an SN with an SF may confuse the shoulder surgeon regarding how to deal with the SSN identification.

## Case presentation

**Figure 1:**
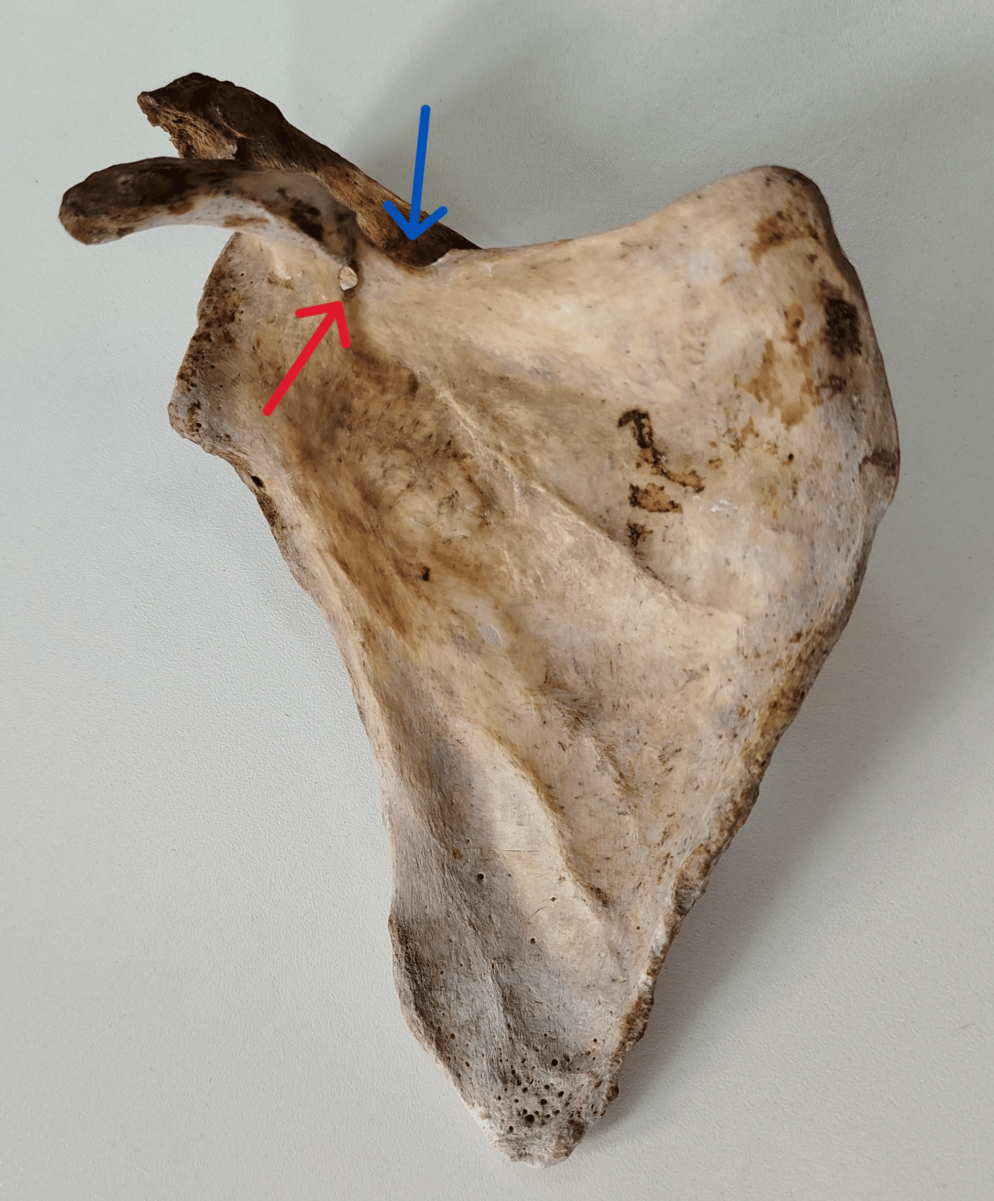
Anterior aspect of the right scapula, showing a shallow SN (blue arrow) and a SF (red arrow) SN: suprascapular notch; SF: suprascapular foramen

**Figure 2:**
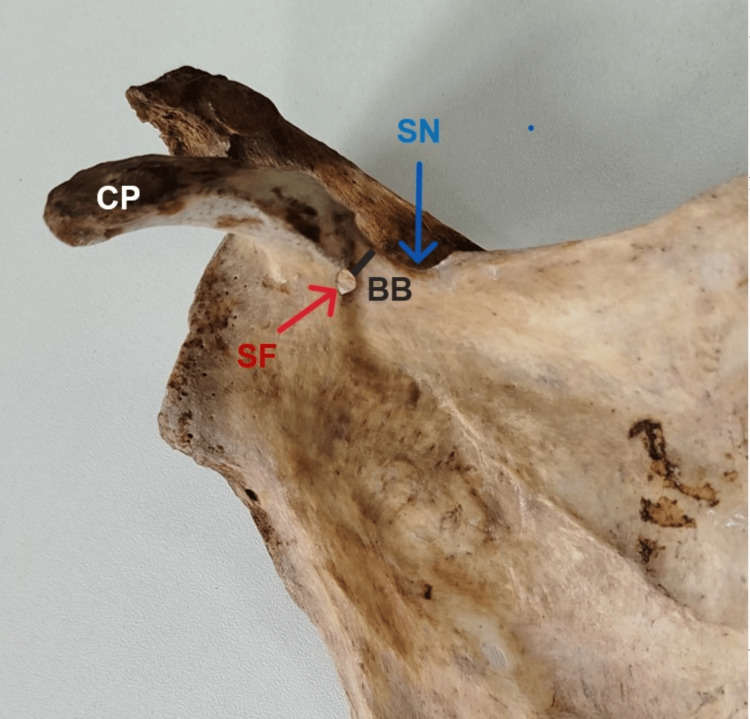
Bony bridge separating SN from SF Anterior aspect showing a shallow SN separated by a BB, which separates SN from SF SN: suprascapular notch; BB: bony bridge; SF: suprascapular foramen; CP: coracoid process

The present study was performed on one dried human right scapula of adult male origin of unknown age obtained from the osteological collection of the Department of Anatomy, Medical School, Aristotle University of Thessaloniki, Thessaloniki. The current scapula displayed a relatively shallow U-shaped SN with the coexistence of an oblique bony bridge of 14 mm in width. The so-formed SSF was oval-shaped, with the vertical diameter being 5 mm and the transverse diameter 3 mm. The foramen was directed obliquely from medial to lateral side, situated at the medial aspect of the root of the coracoid process (Figures 1, 2). The anatomical variation was documented by repeated photographs taken by a digital camera, whereas the measurements were done by a metric digital caliper. The current scapula showed no concomitant lesions or abnormalities in the surrounding area of the detected variant.

## Discussion

The association of SN and SF was mentioned in one case by Hrdicka [2], then in another case by Ticker et al. [6], followed by Natsis et al. [7], who reported three such cases and another two cases in 2008 [8]. Later, Sinkeet et al. [9] found one such case, whereas Polguj et al. [10] observed one bony and two radiological cases of coexisting SN and SF. Ticker et al. [6] attributed the bony bridge of the SN to ossification of an accessory band of the STSL. According to the assumption by Polguj et al. [10], the anterior coracoscapular ligament (ACSL) is ossified, leading to SSF formation along with SN. The ACSL is a ligament placed underneath the STSL. These authors considered that the so-narrowed SSF compresses the SN, especially in shoulder motions, resulting in suprascapular neuropathy [10].

Our case of a shallowed U-shaped SN with coexistence of an SF is added to all the above-mentioned cases. Yavari et al. [11] considered that there is an increased inability for the surgeon to find the SSN in case of an empty SN associated with a very small SF. Moreover, the presence of a double SF has been reported in the literature, attributed to ossification of STSL and ACSL, ossification of a bifid STSL, partial ossification of the trifid STSL, or ossification of the bifid ACSL [12].

Entrapment syndrome or neuropathy of the SSN may occur in 1%-2% of cases of shoulder pain [13]. However, the frequency of suprascapular neuropathy, especially in athletes, has increased to reach 12% up to 33% [14]. Various bony bridges overlying the SSN in the region of the SN may lead to symptoms such as shoulder pain, weakness, and wasting of supraspinatus and infraspinatus muscles with subsequent weakness of abduction and external rotation of the ipsilateral shoulder joint [15]. A completely ossified STSL is related to narrowing of the space of SN, leading to likely entrapment of the SN. Such an ossified STSL, according to Yavari et al. [11], ranged between 0.3% in Eskimos to 30.76% in Brazilians. Moreover, ossified STSL is more frequent in men and on the right side [10], as in our case.

Also, knowledge of the SN morphology is necessary in cases of SNS reinnervation for shoulder reanimation after brachial plexus injuries [11]. Ultimately, a radiologist or surgeon is likely to misdiagnose SF as a lytic lesion of the superior portion of the scapula [16].

## Conclusions

The association of an SN with an SF is very rarely documented in the literature and should be kept in mind by the physician dealing with the shoulder region. The awareness of an SN and SF combination is crucial for the surgeon of the shoulder region and radiologist to properly examine the SN topography in the supraspinatus fossa, interpret a potential SN neuropathy, and differentiate a lytic lesion of the infraspinatus fossa from an SF.
